# Molecular Modeling of ABHD5 Structure and Ligand Recognition

**DOI:** 10.3389/fmolb.2022.935375

**Published:** 2022-06-28

**Authors:** Rezvan Shahoei, Susheel Pangeni, Matthew A. Sanders, Huamei Zhang, Ljiljana Mladenovic-Lucas, William R. Roush, Geoff Halvorsen, Christopher V. Kelly, James G. Granneman, Yu-ming M. Huang

**Affiliations:** ^1^ Department of Physics and Astronomy, Wayne State University, Detroit, MI, United States; ^2^ Center for Molecular Medicine and Genetics, School of Medicine, Wayne State University, Detroit, MI, United States; ^3^ Department of Chemistry, Scripps Florida, Jupiter, FL, United States; ^4^ Center for Integrative Metabolic and Endocrine Research, School of Medicine, Wayne State University, Detroit, MI, United States

**Keywords:** molecular dynamics, docking, structural modeling, ABHD5, lipid droplet, AlphaFold, ligand binding, protein mutation

## Abstract

Alpha/beta hydrolase domain-containing 5 (ABHD5), also termed CGI-58, is the key upstream activator of adipose triglyceride lipase (ATGL), which plays an essential role in lipid metabolism and energy storage. Mutations in ABHD5 disrupt lipolysis and are known to cause the Chanarin-Dorfman syndrome. Despite its importance, the structure of ABHD5 remains unknown. In this work, we combine computational and experimental methods to build a 3D structure of ABHD5. Multiple comparative and machine learning-based homology modeling methods are used to obtain possible models of ABHD5. The results from Gaussian accelerated molecular dynamics and experimental data of the apo models and their mutants are used to select the most likely model. Moreover, ensemble docking is performed on representative conformations of ABHD5 to reveal the binding mechanism of ABHD5 and a series of synthetic ligands. Our study suggests that the ABHD5 models created by deep learning-based methods are the best candidate structures for the ABHD5 protein. The mutations of E41, R116, and G328 disturb the hydrogen bonding network with nearby residues and suppress membrane targeting or ATGL activation. The simulations also reveal that the hydrophobic interactions are responsible for binding sulfonyl piperazine ligands to ABHD5. Our work provides fundamental insight into the structure of ABHD5 and its ligand-binding mode, which can be further applied to develop ABHD5 as a therapeutic target for metabolic disease and cancer.

## Introduction

Lipolysis requires the trafficking and activation of intracellular lipases, such as adipose triglyceride lipase (ATGL, officially known as patatin-like phospholipase domain-containing 2, PNPLA2), to the lipid droplet (LD) surface (Lass et al., 2011; Kintscher et al., 2020). Alpha/beta hydrolase domain-containing protein 5 (ABHD5), also known as comparative gene identification 58 (CGI-58), is a key regulator of the trafficking and activation of ATGL and related members of the patatin-like phospholipase (PNPLA) domain-containing family (Lass et al., 2006; Granneman et al., 2009; Vieyres et al., 2020). Despite its classification as an alpha/beta hydrolase, ABHD5 lacks hydrolase activity owing to the S155N substitution that occurred early in vertebrate evolution (Lass et al., 2006). Instead, ABHD5 evolved structural elements that allow activation of ATGL, as well as a binding site for endogenous and synthetic ligands that regulate interactions with repressor and effector proteins (Sanders et al., 2015). Thus, ABHD5 is a complex protein that contains presently unknown structural elements mediating important functions, including targeting to intracellular LDs, ligand binding, and ATGL activation.

Mutations in the ABHD5 gene cause Chanarin-Dorfman syndrome (Lefevre et al., 2001) wherein the ability of ABHD5 to activate members of the PNPLA family is disrupted and results in disrupted lipid metabolism in numerous organs throughout the body (Hirabayashi et al., 2017; Yang et al., 2019). Previous work has shown the importance of G328 in loss and gain of function assays (Sanders et al., 2017). Recently, a novel mutation of this amino acid in humans (G328E) was reported to produce fatty liver disease (Youssefian et al., 2019). We, therefore, investigated the impact of the G328E mutation in our study. Moreover, endogenous and synthetic ligands bind to ABHD5 protein, which regulates its interactions with protein activators and repressors of lipolysis (Sanders et al., 2015; Rondini et al., 2017).

**FIGURE 1:**
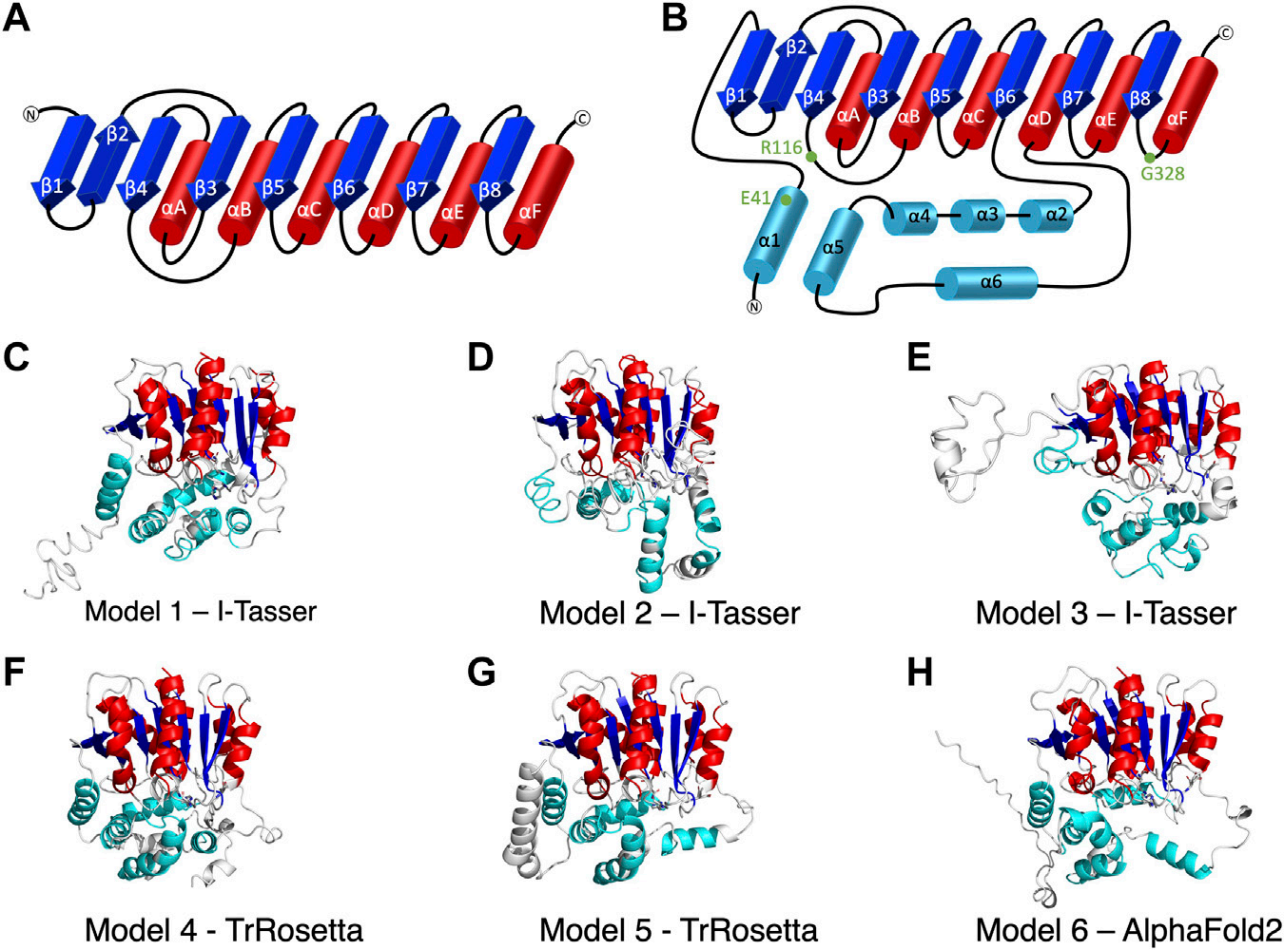
The secondary structure of alpha/beta hydrolase domain-containing 5 (ABHD5). **(A)** The secondary structure diagram of the “canonical” α/β hydrolase fold characterized by six *α* helices (red) and eight *β* strands (blue). The catalytic triad in the α/β hydrolase fold family is made up of 1) a nucleophilic residue (Ser, Cys, or Asp) after β5, 2) an acidic residue after β7, and 3) a conserved His after β8. **(B)** The secondary structure diagram of ABHD5 with two insertion regions (cyan). The first insertion region is an *α* helix (α1), located before β1, and the second insertion region is composed of five *α* helices (α2–α6) between β6 and αD. **(C–H)** The six models of ABHD5 built from different homology modeling tools. Models 1, 2, and 3 are from I-TASSER; Models 4 and 5 are from TrRosetta; and Model 6 is from AlphaFold2. The “canonical” six *α* helices and eight *β* strands for all models are shown in red and blue cartoon representations, respectively. The six insertion *α* helices are shown in cyan. The secondary structures are defined as: insertion α1 (residues 33–46), β1 (residues 53–59), β2 (residues 64–71), β3 (residues 80–84), αA (residues 89–103), β4 (residues 106–111), αB (residues 126–143), β5 (residues 149–154), αC (residues 157–171), β6 (residues 172–181), insertion α2 (residues 198–207), insertion α3 (residues 221–229), insertion α4 (residues 232–240), insertion α5 (residues 245–257), insertion α6 (residues 259–270), αD (residues 278–288), β7 (residues 292–298), αE (residues 305–314), β8 (residues 319–325), and αF (residues 331–351). Asn155, Asp303, and H329 are drawn in sticks with the carbon atoms in white, nitrogen atoms in blue, and oxygen atoms in red. The hydrogen atoms are not shown.

Because of the importance of lipolysis, efforts have been made to determine ABHD5 structure. NMR experiments revealed the structure and flexibility of the tryptophan-rich N-terminal peptide (residues 10–43) of ABHD5 (Boeszoermenyi et al., 2015). Even though the experimental structure of the entire protein is still unknown, ABHD5 is considered to share the 3D features of the alpha/beta hydrolase fold superfamily. The “canonical” alpha/beta hydrolase fold (Ollis et al., 1992) and the variations inserted among these folds (Nardini and Dijkstra, 1999) have been suggested for the members of the ABHD family (Figure 1A). To enhance the structural understanding of ABHD5 activation of ATGL, a homology model of ABHD5 was built by Modeller (Sanders et al., 2017). The model identified R299, G328, and D334 as critical for ATGL activation, which was validated experimentally in both gain- and loss-of-function assays. In this model, G328 was suggested to interact with phospholipids to provide favorable interactions of R299 and D334 (Sanders et al., 2017).

Homology or comparative modeling is the most efficient computational method to predict 3D protein structures using 1D protein sequence data (Hameduh et al., 2020). This modeling approach is based on two assumptions: 1) the 3D structure of a protein is uniquely determined by its amino acid sequence, and 2) during evolution, the changes in the structure are much slower than the changes in the sequence; hence similar sequences adopt similar physical structures. Traditionally, in homology modeling, the structure of the target protein is determined based on another experimentally derived structure, termed template. The 3D structure of the target protein is then built based on the alignment with the chosen template. For example, I-TASSER is one of the broadly used servers (Roy et al., 2010; Yang et al., 2015). With years of effort, homology modeling has become a major approach in structural prediction, benefiting from the ever-growing number of high-resolution experimental structures deposited in the protein data bank (PDB) and a multitude of new algorithms that improve target-template alignment. In recent years, deep learning-based methods have provided an innovative approach to structural modeling, even when no similar structures are available. The AlphaFold1 algorithm, developed by Alphabet/Google DeepMind, was first introduced in 2018 (Wei, 2019). The method applies convolutional neural networks to predict inter-residue distances, which inspired other deep learning-based methods such as trRosetta in protein structural prediction (Du et al., 2021). The new version, AlphaFold2, released in 2021 (Jumper et al., 2021; Jumper and Hassabis, 2022), uses an innovative network architecture to model atomistic positions with an average success rate greater than 90% (Marx., 2022).

Molecular dynamics (MD) simulations have been successful in numerous biomolecular simulations at the atomic level since they were introduced in 1977 (McCammon et al., 1977). However, despite recent advances, conventional MD (cMD) simulations of biomolecules remain limited to timescales of hundreds of nanoseconds to tens of microseconds, whereas most biological processes take place over milliseconds and longer timescales. To overcome this limitation, two types of enhanced sampling methods were developed. The methods, such as umbrella sampling (Torrie and Valleau, 1977) and metadynamics (Laio and Parrinello, 2002), require the definition of a set of collective variables. However, the algorithms, such as replica-exchange dynamics (Sugita and Okamoto, 1999), accelerated MD (Hamelberg et al., 2004), and Gaussian accelerated MD (GaMD) (Miao et al., 2015), obviate this requirement. GaMD is an enhanced sampling approach in which users may access large biomolecule conformational changes within hundreds of nanoseconds (Miao et al., 2015; Wang et al., 2021). By adding a harmonic boost potential to the original energy surface of the system, users do not need to provide any information about the boost reaction coordinates before executing the simulation, which avoids the simulation bias from pre-defined variables. GaMD has been successfully used in protein-ligand binding, protein folding, and ion channel dynamics studies (Wang et al., 2021). It has been applied in various types of biosystems, such as G-protein-coupled receptor (Miao and McCammon, 2018), HIV protease (Miao et al., 2018), CRISPR-Cas9 (Nierzwicki et al., 2021), ACE2 receptor (Bhattarai et al., 2021), androgen receptor (Zhan et al., 2021), and p38 kinase (Huang, 2021).

**FIGURE 2:**
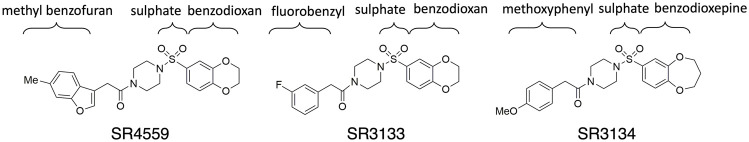
The 2D sketches of the three sulfonyl piperazine (SPZ) ligands.

In this work, we aimed to construct the atomistic structure of ABHD5 to uncover its dynamics and ligand recognition using newly available computational tools together with experimental validation. First, traditional and deep learning-based homology modeling methods were used to build multiple 3D models for ABHD5. Then, the functional activation was used to select potential ABHD5 structures. The dynamics of ABHD5 under physiological conditions were studied using microsecond-long GaMD simulations. The ABHD5 systems with point mutations were also built and modeled. Finally, we reveal the binding mechanism of synthetic ligands to ABHD5, focusing on one of the independent chemical scaffolds, sulfonyl piperazines (SPZs) (Figure 2) that was shown to promote the lipase-activating state of ABHD5 by disrupting its interaction with perilipin 1 (PLIN1) and perilipin 5 (PLIN5) repressors (Sanders et al., 2015). Our work provides a framework for ABHD5 structural modeling and insights into its interaction surface with ligands and related partners, which helps the understanding of ABHD5 functional evolution and lipase regulation.

## Materials and Methods

### Modeling Systems

This work focuses on the ABHD5 protein from *Mus musculus*. The protein sequence was obtained from UniProt with the entry ID: Q9DBL9. Three SPZ ligands, SR-01000604559 (CID: 2674365), SR-03000003133 (CID: 4827533), and SR-03000003134 (CID: 25701322), were investigated in this study. The abbreviations SR4559, SR3133, and SR3134 are applied to the ligands in this paper (Figure 2). The Maestro software was used to build the ligand structures for later docking and MD simulations.

### Structural Modeling

I-TASSER (Yang et al., 2015), TrRosetta (Du et al., 2021), and AlphaFold2 (Jumper and Hassabis, 2022) were the three programs used to predict ABHD5 structures. I-TASSER requires a structural template, which can be chosen by the software (default mode) or provided by the user. Both TrRosetta and AlphaFold2 employ a deep learning algorithm for protein structure prediction and do not need a user input template. TrRosetta requires only the protein sequence, and the predicted structures by AlphaFold2 are available on AlphaFold Protein Structure Database. To obtain the structural templates for I-TASSER, the blastp-suite of Protein BLAST (Zhang and Madden, 1997; Altschul et al., 1998; Madden et al., 2019) and LALIGN (Madeira et al., 2019) programs were applied to search for potential templates according to the structural data presented in PDB. A total of five templates were selected, PDB code: 6I8W, 3NWO, 3SK0, 1S8O, and 6NY9. The first four templates have the highest overall alignment scores to ABHD5 (Supplementary Table S1). The last template, 6NY9, was selected as it is a high resolution (1.66 Å) X-ray crystallography structure of ABHD10, a protein in the same family and from the same organism (Cao et al., 2019). Using I-TASSER, we performed six calculations, including the default mode and five calculations for each template we selected above, which reports 30 models in total. TrRosetta offered five models, and AlphaFold2 provided only one model. To select models for later studies, we used the g_cluster program, an RMSD-based clustering method, in GROMACS 2021.2 (Pronk et al., 2013) to cluster the resulted models. The program reported the six most representative ABHD5 structures, shown in Figures 1C–H.

### Simulation System Setup

To build hydrogen atoms on ABHD5, the protonation states of ABHD5 were assigned by the Adaptive Poisson Boltzmann Solver (APBS) webserver (Jurrus et al., 2018). We applied the Amber18 package (Case et al., 2015) for the GaMD simulation setup and production simulations with an efficient GPU implementation. The Amber ff14SB (Maier et al., 2015) and General Amber Force Field (GAFF) were applied to the protein and ligand, respectively (Ozpinar et al., 2010). Before solvation, energy minimization was performed on the hydrogen atoms, protein side chains, and the entire system for 500, 5,000, and 5,000 steps, respectively. The systems were then solvated in TIP3P water molecules (Jorgensen et al., 1983; Izadi and Onufriev, 2016) with ∼12 Å between the box edge and the solutes to create a rectangular box. A salt concentration of 150 mM NaCl was added to the system (Machado and Pantano, 2020) using the ion parameters developed by Joung and Cheatham (2008). Additional minimizations of the water and ion molecules and the entire system (including water, ions, and protein) were performed for 2 and 10 ps, respectively. The equilibration of the system started from the solvent equilibration for 100 ps, then the complex system was gradually heated to 50, 100, 150, 200, 250, and 300 K for 10 ps at each temperature using the isochoric-isothermal (NVT) ensemble. To ensure the system reached equilibrium, a 5.0 ns cMD simulation was further performed at 300 K using the isobaric-isothermal (NPT) ensemble. The time step of the MD simulations was 2 fs. Periodic boundary conditions were applied for the simulation systems, and long-range electrostatics were accounted for using the particle mesh Ewald summation method (Essmann et al., 1995) with a cutoff of 12 Å. Bonds containing hydrogen atoms were restrained using the SHAKE algorithm (Krautler et al., 2001). The Langevin thermostat with a damping constant of 2 ps^−1^ was turned on to maintain a temperature of 300 K (Loncharich et al., 1992).

### GaMD Simulations

GaMD (Miao et al., 2015) is an enhanced sampling method that can perform aggressive sampling of a biomolecule. By adding a harmonic boost potential (*∆V*) on the original potential energy surface (*V*), the modified potential (V*) can be written as $V^{\text{*}}\left(\vec{r}\right)=V\left(\vec{r}\right)+\Delta V\left(\vec{r}\right)$

$$\text{Δ}V\left(\vec{r}\right)=\;\{\begin{array}{cc} \frac{1}{2}k{\left(E-V\left(\vec{r}\right)\right)}^{2}, & \;if\;V\left(\vec{r}\right)<E\\ 0, & if\;V\left(\vec{r}\right)\geq E \end{array}$$ (1)

where *k* is the harmonic force constant and *E* is the threshold energy that should be lower than the system potential (*V*). To ensure that the boost potential does not alter the shape of the original potential surface, the threshold energy needs to satisfy the following relation:

$$V_{max}\leq E\leq V_{min}+\frac{1}{k}$$ (2)

where *V*_*max*_ and *V*_*min*_ are the system maximum and minimum potential energies, respectively.

To start, a 2-ns cMD simulation was executed to collect the potential statistics, such as *V*_*max*_ and *V*_*min*_, for calculating GaMD acceleration parameters. Then, we performed a 1-ns GaMD simulation with applied boost potential but no updating on *V*_*max*_ and *V*_*min*_ values. The second GaMD simulation was carried out with the updated boost potential for 50 ns. Finally, we performed 1,000 ns production GaMD simulations with a fixed boost potential for all systems. The upper bound for the boost acceleration was selected for all simulations (iE = 2). The average and standard deviation of the system potential energies were calculated every 500 ps. The boost potential was added to both the dihedral energy and the total potential energy. The upper limit of the standard deviation of the potential energy was set to 6.0 kcal/mol for both the dihedral and total potential energy terms. We saved the resulting trajectories every 10 ps for analysis. Note that, GaMD modifies the potential energy surface without considering the entropic contribution. However, the entropic effect would not affect the overall calculation in this study.

We performed 1-µs GaMD simulations for six wild-type ABHD5 models. Because Model 6 was selected as the most representative ABHD5 model, additional four 1-µs GaMD simulations were executed. We also performed 1-µs GaMD simulations for the three ABHD5 mutants (E41A, R116N, and G328E) and three ABHD5-ligand complexes.

### Post-GaMD Analysis

All simulation trajectories were visualized by the VMD program (Humphrey et al., 1996). The CPPTRAJ program (Roe and Cheatham, 2013) from the Amber18 package (Case et al., 2015) was used to analyze the root mean square fluctuation (RMSF), residue-residue correlation, and hydrogen bonds. The RMSF was used to measure the average fluctuation of the Cα atom of a specific protein residue over time. The hydrogen bonds were considered when the donor-acceptor distance was less than 3.5 Å, and the donor-hydrogen-acceptor angle was less than 30°. The amino acids in the ligand binding pocket were defined as the residues that are within 3.5 Å of the ligands, and the occupancy is over 75% of 1-µs GaMD trajectories. To obtain the representative conformation from the GaMD trajectories of the six models, the g_cluster program from GROMACS 2021.2 (Pronk et al., 2013) was used to cluster the 1-µs trajectory for each model into 10 conformations. The most representative conformation of each model was reported. The VMD (Humphrey et al., 1996) and PyMOL, programs were used to create images for the publication.

### Ligand Docking

We applied the g_cluster program from GROMACS 2021.2 (Pronk et al., 2013) to cluster the structural ensemble from the 1-μs GaMD trajectories of the ABHD5 protein built by AlphaFold2 into 10 representative conformations. Because the protein is restrained during docking simulations, to avoid the structural bias, these 10 conformations were used for ligand docking. AutoDock Vina (Trott and Olson, 2010; Eberhardt et al., 2021) was applied to dock three Scripps Research (SR) ligands shown in Figure 2. The 3D structures of all ligands were prepared using Schrödinger Maestro software. The AutoDock Tools (ADT) (Morris et al., 2009) of the MGLTools (Forli et al., 2016) was used to prepare the proper file formats (pdbqt) for the ligands and the protein conformations and to determine the docking box sizes, which were set to 26, 28, and 30 Å. The docking box center was selected near the center of the protein and the exhaustiveness value was set to 64. For each ligand system, we performed a 1-µs GaMD simulation on the reported ABHD5-ligand complex structure with the best docking score.

### Cell Culture, Imaging, Scoring, and Luciferase Complementation

ABHD5-dependent activation of ATGL was performed as described previously (Sanders et al., 2017). Briefly, Cos7 cells plated on coverslips in 12-well dishes were transfected with 0.5 μg each/well of mCherry-tagged ABHD protein, PLIN5, or ATGL using Lipofectamine and Plus reagent (Invitrogen) as described by the manufacturer. Cells were then lipid-loaded for 16–20 h with 200 μM oleic acid, then fixed with 4% paraformaldehyde. Images were acquired with an Olympus IX-81 microscope equipped with a spinning disc confocal unit. Images were captured using a 60x, 1.4 NA objective and a Hamamatsu ORCA Flash cooled CMOS camera. The following Chroma filter sets were used for the indicated fluorophores: mCherry, 41043; EYFP, 31044; ECFP, 41028. LD scoring was performed by an investigator blinded to transfection conditions. For each transfection condition in each experiment, 25 or more cells visibly expressing all three proteins were scored. Mutant ABHD5 proteins were made using standard molecular biological methods, and all PCR-derived proteins were confirmed by sequencing. ABHD5 proteins are from mice, and the numbering of amino acids refers to the mouse protein unless indicated otherwise. PLIN5 and ATGL were also from the mouse.

ABHD5 ligand binding was assessed by ligand-induced inhibition of luciferase complementation between ABHD5 and PLIN5, as previously described (Granneman et al., 2007). Briefly, cell lysates were prepared from 293T cells that were transiently transfection with ABHD5 or PLIN5 fused to the C- or N-terminal fragments of G. princeps luciferase, respectively. Lysates were mixed together in the presence of indicated concentration of ABHD5 ligands or DMSO (control) for 4 h at room temperature, after which coelenterazine substrate was added and the resulting luminescence was read in a Clariostar multiplate reader. Ligand affinity (IC_50_ values) was determined by nonlinear regression using GraphPad software.

## Results and Discussion

### ABHD5 Structures

We report six potential ABHD5 structures (Figures 1C–H; Supplementary Text S1). Models 1, 2, and 3 were selected from the resulting structures created by I-TASSER, a template-based homology modeling method. Models 4 and 5 were built using TrRosetta, and the structure obtained from AlphaFold2 is shown as Model 6. Both TrRosetta and AlphaFold2 are deep learning-based methods for protein structure prediction. Despite using different computational methods in constructing these six ABHD5 models, all structures follow the “canonical” alpha/beta hydrolase fold (Ollis et al., 1992; Mindrebo et al., 2016), where the protein is composed of six *α* helices, eight *β* sheets, and two insertion regions—an *α* helix (α1) near the N-terminal before β1 and the helical insertions (α2–α6) between β6 and αD (Figure 1B). In the six models, the three catalytic triad residues, N155, H329, and D303, are all in proximity to each other. Although the lipase substrate/ligand site is preserved, it is not an active site as ABHD5 lacks hydrolase activity (Lass et al., 2006).

The largest variations among the six models come from the N-terminal (residues 1–52) and the insertion region between β6 and αD (residues 198–270). Our 1-µs GaMD simulations reveal that both the N-terminal and the insertion regions display high RMSD and RMSF values (Supplementary Figures S1B,C, S2), indicating these areas are flexible in the ABHD5 protein. Although the alpha helices predicted by the homology modeling in these regions maintain along the GaMD simulations, the loops connecting the helices fluctuate. This is consistent with the report from AlphaFold2 that the structural modeling on these areas has the lowest confidence. In Models 1, 4, 5, and 6, the N-terminal residues form *α* helices. Models 1, 4, and 6 show a helix from residues 33–46, while Model 5 contains three helices, residues 1–19, 21–25, and 33–46. These α helices are all close to the bulk of the protein in Model 5. In Model 2, although the N-terminal does not form a secondary structure, it still stays close to the rest of the protein. However, the N-terminal in Model 3 is different from other models, in which only a short helix (residues 16–26) is present, and the N-terminus does not form interactions with other parts of the protein (Figure 1E). The results from both GaMD and homology modeling agree well with the early NMR finding (Boeszoermenyi et al., 2015) that the N-terminal peptide is very flexible and may not form a stable secondary structure. The most representative conformation of each model from the GaMD simulations is shown in Supplementary Figure S3.

### Validation and Differentiation of ABHD5 Models

**FIGURE 3:**
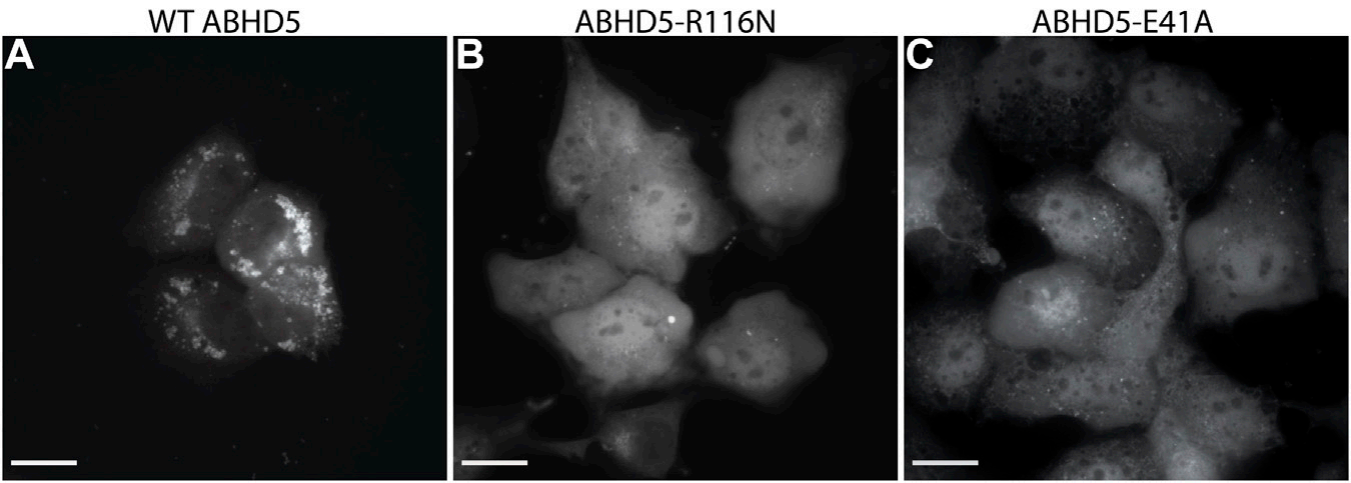
Fluorescence imaging reveals the importance of R116 and E41 in the basal localization of ABHD5. **(A)** Wild-type (WT) ABHD5-mCherry localizes to the LDs within COS7 cells. Either the **(B)** R116N or **(C)** E41A mutations demonstrate a cytosolic distribution of ABHD5-mCherry and inhibited basal LD targeting in the absence of ATGL and PLIN5 expression. **(A–C)** The scale bars represent 20 µm.

To validate the ABHD5 models created from homology modeling, we first executed point mutation experiments and GaMD simulations. A critical step in the activation of lipolysis is the association of ABHD5 to lipid membranes, and we found that a point mutation, R116N, is specifically defective in basal membrane binding (Figure 3). We hypothesized that R116 stabilizes an amphipathic helix that facilitates the membrane binding of ABHD5. To gain deeper insights into the structural basis, we examined the residue-residue interactions near R116 in the six models. Except in Models 2 and 3, R116 forms interactions with E41. The distance between the sidechain oxygen of E41 (OE1/2) and the sidechain nitrogen of R116 (NH1/2) is 2.77, 10.92, 34.21, 4.70, 4.96, and 2.72 Å for Models 1–6, respectively. We next examined the dynamics of all models in solution by GaMD simulations. In Models 1, 4, 5, and 6, our 1-µs simulations reveal that R116 forms stable electrostatic interactions with E41, within an α-helix encompassing residues 33–46, and R116N mutation disrupts the interaction between R116 and E41 and its associated helix. However, no R116-E41 interactions are found in Models 2 or 3. To evaluate if the R116-E41 interactions affect ABHD5 membrane binding, we performed a point mutation experiment on E41. We found that the E41A mutation of ABHD5 phenocopies the R116N mutant—both mutants were defective in basal membrane binding (Figure 3). The experimental results support the hypothesis from Models 1, 4, 5, and 6 that the interactions between R116 and E41 play a key role in stabilizing the ABHD5 structure and promoting an extended amphipathic helix to penetrate the phospholipid tails and bring about membrane binding. Taken together, Models 2 and 3 present unlikely structures of ABHD5 as the interactions between E41 and R116 are missing.

To further differentiate Models 1, 4, 5, and 6, we examined the interactions of R299, G328, and D334, which are conserved residues necessary for mediating PNPLA2 activation (Sanders et al., 2017). We hypothesized that a stable interaction of R299, G328, and D334 would occur in more probable models of ABHD5. In Models 4, 5, and 6, the interactions of R299, D328, and D334 are stable during 1-µs GaMD simulations. However, in Model 1, R299 moves away from G328 and D334, destabilizing their interactions during the GaMD simulation (Supplementary Figure S4A).

In Models 4 and 5, the N-terminal peptide (residues 1–32) either interacts with the insertion helices, α2, α3, and α6, or makes direct contact with D334 (Supplementary Figures S4F,G), both of which are not a preferred structure for an LD-bound conformation of ABHD5. It has been hypothesized that the N-terminal peptide forms interactions with the phospholipids on the LD surface (Boeszoermenyi et al., 2015), so the N-terminal peptide should be near E41 and R116. Although the goal of this work is not to obtain a membrane-bound structure of ABHD5, identifying a protein structure consistent with the membrane-bound conformation will assist in further experimental and computational studies (Sanders et al., 2015; Rondini et al., 2017). Because the N-terminal peptide in ABHD5 is highly flexible, further simulations of Models 4 and 5 may reveal changes to the peptide position that are consistent with the membrane-binding conformation. However, to save computational efforts, we chose Model 6 for the following mutation and ligand binding studies because, in Model 6, we found that 1) E41 forms interactions with R116 that stabilize the N-terminal amphipathic helix, 2) the interactions of R299, G328, and D334 are stable, and 3) the N-terminal peptide is closer to the protein-membrane binding surface.

### ABHD5 Protein Dynamics

**FIGURE 4:**
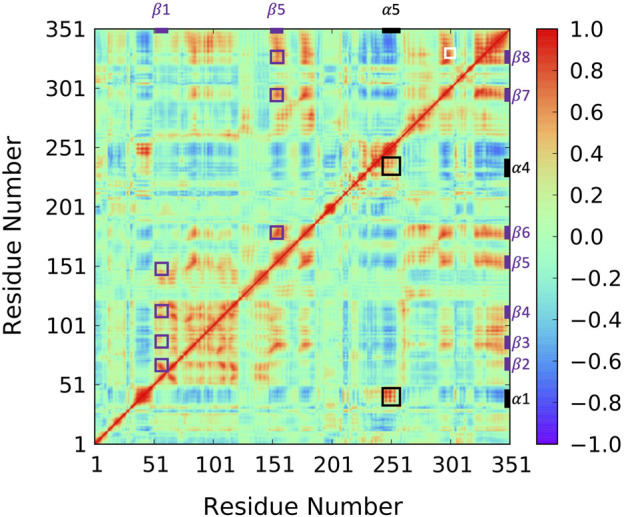
Correlation map for 1-µs GaMD trajectory of Model 6. The correlation between β1 and β2–β5 as well as the correlation between α5 and β6–β8 are highlighted using purple boxes. The correlation between insertion helix α5 and insertion helices α1 and α4 are highlighted with black boxes. The correlation formed by R299, G328, and D334 is highlighted by a white box.

To reveal ABHD5 protein dynamics and equilibrate the structure created from homology modeling, we performed five replicas of 1-µs GaMD simulations on Model 6 created by AlphaFold2. Our simulations show that the *β* strands (β1–β8) form strong interactions and correlations with each other (Figure 4 purple labels), stabilizing the overall protein folding. The GaMD simulations also refine some helical structures, such as αD, which was reported with low confidence by the AlphaFold2 algorithm (Jumper and Hassabis, 2022). In this canonical alpha/beta hydrolase fold, the eight *β* strands and the six *α* helices demonstrate less fluctuations as indicated by the RMSF calculations (Supplementary Figure S2). However, the N-terminal (residues 1-52 including α1) and the region composed of insertion helices (residues 198–270 including α2-α6) are highly flexible, which is also the main variance within other alpha/beta-hydrolase proteins. The α5 insertion helix shows correlations with the α1 and α4 insertion helices (Figure 4 black labels). No correlations were found between other insertion helices, suggesting that the motions of the secondary structures in the insertion area are mostly independent.

Since the mutation of R299 and G328 disrupt the ATGL activation by ABHD5 (Sanders et al., 2017), we closely examined the interactions near these two residues. Our model shows that R299 and G328 are in the vicinity of D334. The three residues, R299, G328, and D334, are located on the protein surface exposed to the solvent molecules. The residues form a stable hydrogen bond network and move together during the simulation (Figure 4 white label; Supplementary Figure S4). These results are consistent with the previously reported homology model created by Modeller (Sanders et al., 2017; Tseng et al., 2022).

### ABHD5 Mutations

Mutations of the ABHD5 protein can alter its membrane binding, protein binding, ligand binding, lipolysis activation, and consequently its function. In this study, three new ABHD5 mutations were identified—E41A and R116N affect the ABHD5 membrane binding (Figure 3), and G328E suppresses the ability of ABHD5 to activate ATGL (Supplementary Figure S5). These mutations alter the local interactions with nearby residues and further restrain the protein function.

**FIGURE 5:**
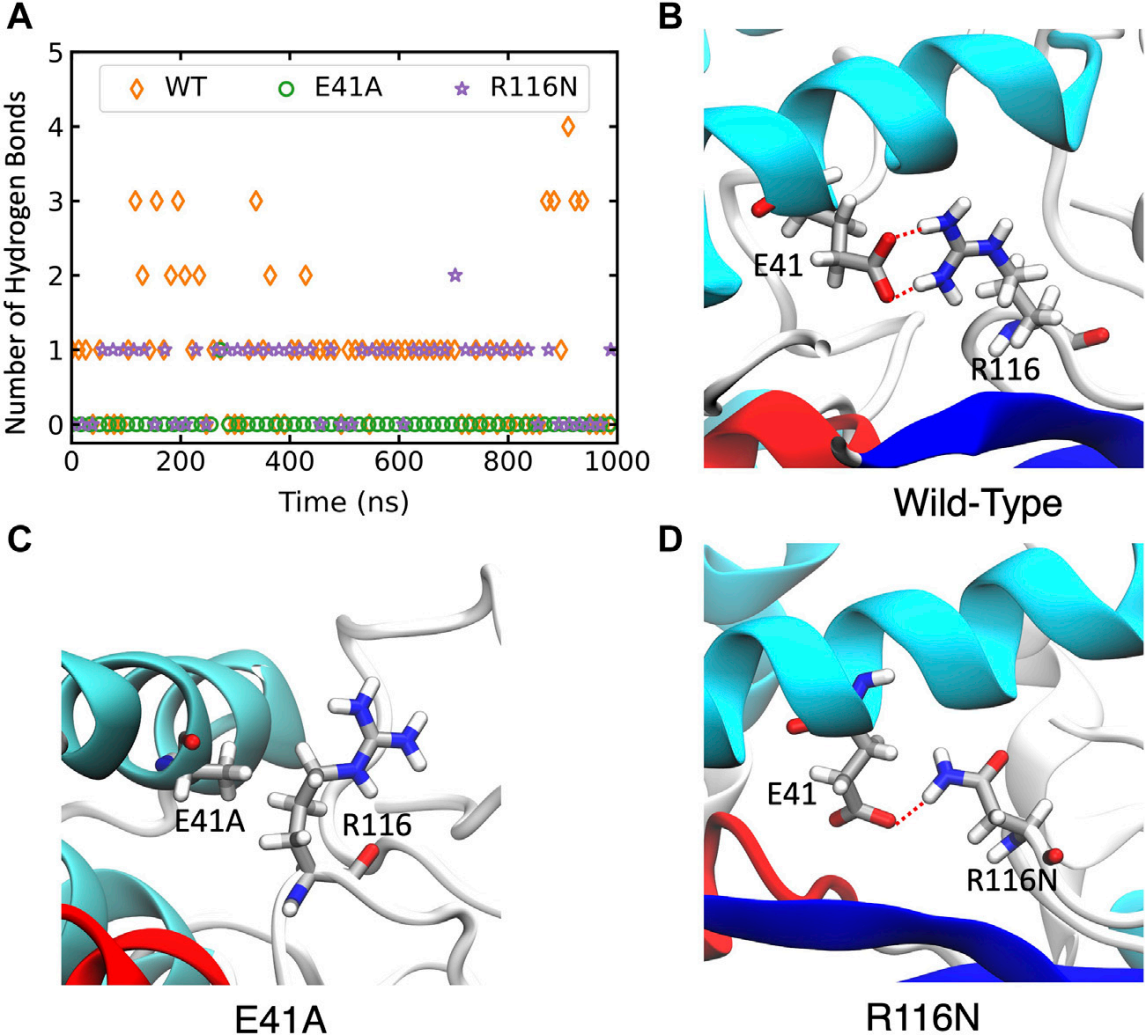
The changes of hydrogen bonds between residues 41 and 116 in the wild-type (WT) ABHD5 and its mutants. **(A)** The number of hydrogen bonds between residues 41 and 116 over the course of 1-µs GaMD trajectories. The changes in hydrogen bonds of the WT, E41A, and R116N systems are indicated by orange, green, and purple, respectively. **(B–D)** The GaMD snapshots of the WT, E41A, and R116N system.

In wild-type ABHD5, E41 forms hydrogen bonds with R116, K38, and K54 (Supplementary Figure S6A). The hydrogen-bonding network between these four residues stabilizes the secondary structure of α1, β1, the loop between β4 and αB, and a cavity that could facilitate the binding of negatively charged phospholipid heads as the binding site is composed of the positively charged K38, K54, and R116. The E41A mutation disrupts the hydrogen bonding network (Figure 5; Supplementary Figure S6B). Without this hydrogen bonding, the sidechains of K54 and R116 rotate away from the cavity and no longer move in concert (Supplementary Figure S7), which may contribute to the reduced membrane binding of E41A. With the E41A mutation, the smaller alanine sidechain contributes to the increased distance between E41A and R116, which results in structural changes of nearby insertion helices. For example, the α5 insertion moves closer to insertion α1. This motion of the insertion α5 alters the mobility of the neighboring insertion helices, α3, α4, and α6 (Supplementary Figure S8). Although the E41A mutation does not alter the overall folding of the eight canonical *β* strands, the correlation within the *β* strands reduces significantly (Supplementary Figure S7).

The R116N ABHD5 reveals very similar experimental phenotype and molecular dynamics results to the E41A mutant. Although E41 can still form hydrogen bonds with R116N, the occupancy of hydrogen bonding in the 1-µs GaMD simulation reduces from 71.7% to 56.3%, leading to deformation of the cavity formed by K38, E41, K54, and R116N. We hypothesized that the misorientation of the K38, E41, K54, and R116 sidechains disturbs phospholipid binding and membrane targeting (Supplementary Figure S6C). The R116N mutation alters the motions of neighboring insertion helices, similar to the E41A mutation.

In wide-type ABDH5, G328 is spatially close to R299 and D334 while both the R299 sidechain and the G328 mainchain form stable hydrogen bonds with the D334 sidechain (Supplementary Figure S9A). Both R299 and G328 are situated at loops—R299 is at the loop between β7 and αE, and G328 is at the loop between β8 and αF, whereas D334 is in the αF helix. The electrostatic interactions among the three residues connect the two loops and the αF helix, stabilizing the loop conformation. Five independent GaMD simulations of the wild-type ABHD5 show a pocket formed by the insertion helices, α2 and α4, and the two loops containing R299, G328, and D334 (Supplementary Figure S9C). The pocket could accommodate the binding of ABHD5 protein partners. In humans, G328E mutation results in neutral lipid storage disease (Youssefian et al., 2019), so it is of interest to determine how this mutation alters the shape of this critical pocket. We found that the positively charged sidechain of G328E forms strong electrostatic interactions with R299, eliminating the interactions between R299 and D334 and altering the conformation of the loop between β7 and αE (Supplementary Figure S9B). In addition, the charged sidechain of G328E also forms hydrogen bonds with V198 near the insertion helix α2, which further alters the structure of insertion α2 and results in closing of the pocket (Supplementary Figure S9D). For example, the distance between G328Cα and V198Cα reduces after the mutation (Supplementary Figure S10). In the G328E GaMD simulation, the RMSF measurement reduces in the region between amino acids 300 and 340 (Supplementary Figure S2), indicating that the reduced flexibility of the protein may affect the ATGL activation.

### ABHD5 Ligand Complexes

**FIGURE 6:**
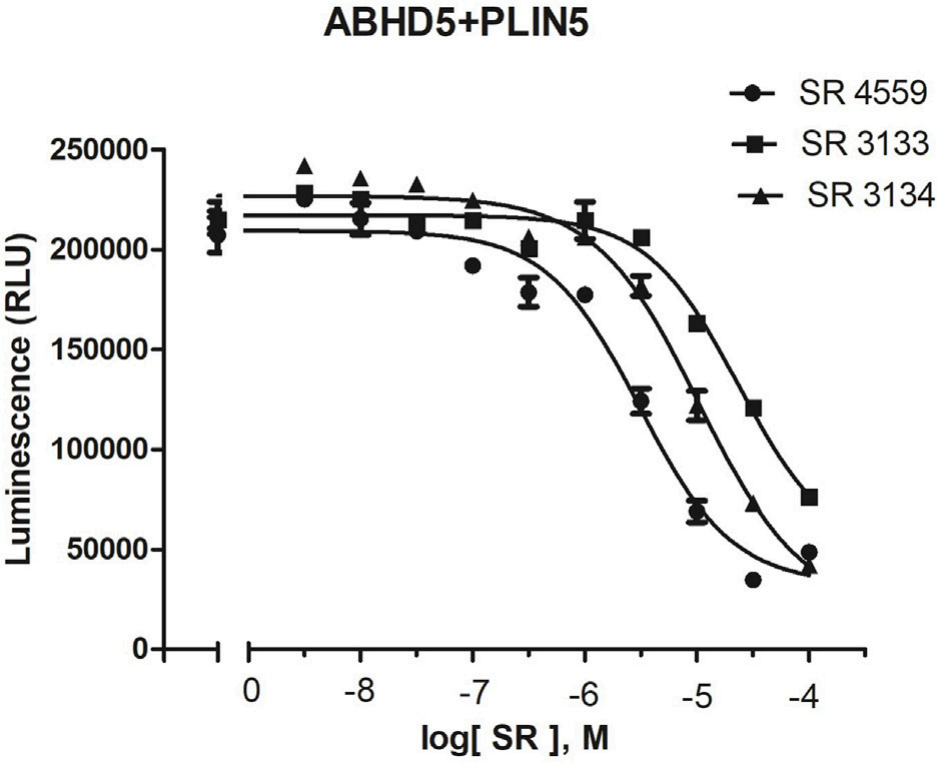
The binding of SPZ ligands to ABHD5 was assessed by dose-response curves of ABHD5-PLIN5 binding. ABHD5-PLIN5 binding was assessed by luciferase complementation assays of Cos7 lysates. Each ligand has micromolar IC_50_ values with specific binding to ABHD5 (Sanders et al., 2015).

To reveal the dynamics of ABHD5-ligand complexes, we explored the binding mode of three synthetic SPZ ligands, namely SR4559, SR3133, and SR3134. The three ligands are structurally analog with a similar 2D sketch (Figure 2). Both SR4559 and SR3133 have a benzodioxan and sulphate functional group. SR4559 includes a methyl benzofuran group, while SR3133 has a fluorobenzyl group. SR3134 includes a benzodioxepine group instead of a benzodioxan group of SR4559 and SR3133. SR3134 also has a sulphate group and displays a similar sketch to SR3133. Although some computational techniques, such as attach-pull-release (Velez-Vega and Gilson, 2013), confine-and-release (Cacelli and Prampolini, 2007), and BFEE2 (Fu et al., 2022) may estimate ligand binding affinities *in silico*, we measured the ABHD5-SPZ ligand binding by experiments. The results show that SR4559, SR3133, and SR3134 dissociate the ABHD5-PLIN5 complexes in Cos7 cell lysates with IC_50_ values of 3.44 ± 0.50 × 10^−6^ M, 1.59 ± 0.66 × 10^−5^, and 8.90 ± 1.84 × 10^−6^ M, respectively (Figure 6), indicating that all three ligands bind ABHD5.

**FIGURE 7:**
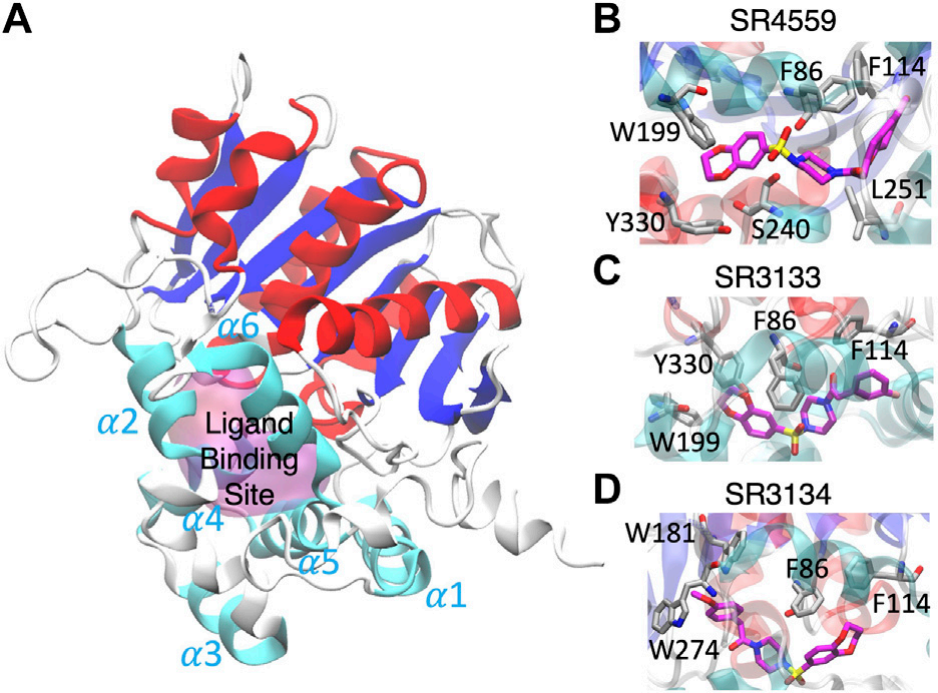
The ligand binding modes of SPZ ligands. **(A)** SPZ ligands bind to the pocket (magenta) formed by insertion helices, α2-α6 (cyan). Red and blue indicate the canonical *α* helices and *β* strands, respectively. **(B–D)** reveal the residues (carbons in gray) interacting with SR4559, SR3133, and SR3134 (carbons in magenta), respectively.

Our docking and GaMD simulations show that the three ligands are located at the pocket formed by the insertion helices, α2-α6 (Figure 7A; Supplementary Text S1). The region of the insertion helices is typically flexible and challenging in protein structural prediction, thus the accuracy of the predicted ABHD5 structure directly corresponds to the docking results. The RMSF values for the residues 220–250 significantly reduce in the three ligand-bound models compared to the apo model (Supplementary Figure S11), suggesting that the ligand binding restrains the protein motion. Besides, each SPZ analog interacts with similar residues (F86, G87, F114, L203, A206, N211, Y238, S240, I251, N255, and E262) in the binding pocket (Supplementary Table S2).

Although the residues contributing to ligand binding are similar, the detailed interactions are different for each ligand. In the SR4559 binding, the methyl benzofuran group forms nonpolar interactions with F86, F114, and L251, and the benzofuran functional group interacts with W199, S240, and Y330 (Figure 7B). Although SR3133 displays a similar binding mode to SR4559, fewer contacts with protein residues were found. For example, the fluorophenyl group interacts with F86 and F114, and the benzofuran functional group interacts with W199 and Y330 (Figure 7C). SR3134 presents a different binding mode. The benzodioxepine group interacts with F86 and F114, while the methoxyphenyl functional group binds deeply into the pocket formed by W181 and W274 (Figure 7D).

## Conclusion

In this work, we employed traditional and deep learning-based homology modeling tools to model the structure of the ABHD5 protein. The ABHD5 structures reported from the deep learning-based modeling, including TrRosetta and AlphaFold2, are most consistent with experimental analysis of E41, R116, R299, G328, D334, and the N-terminal mutants. We therefore selected the AlphaFold2 model for mutation and ligand docking studies, as the orientation of the N-terminal peptide is close to the residues required for the ABHD5 membrane binding. Our structural modeling and dynamics simulations show that the canonical α helices and *β* strands of the ABHD5 protein are highly stable. The main variance in the structure originates from the insertion of helical regions, which are correlated to essential ABHD5 functions, such as membrane and ligand binding. The E41A and R116N mutations disturb the ABHD5 membrane binding by disrupting the hydrogen bonding network of several nearby lysine residues (K38 and K54). The mutation of G328E changes the electrostatic interactions of the surrounding residues, thereby affecting the activation of ATGL. Our study also reveals the ligand binding modes of three SPZ ligands to ABHD5. The results show that the SPZ ligands bind stably in the hydrophobic pocket formed by the insertion helices, α2-α6.

## Data Availability

The original contributions presented in the study are included in the article/Supplementary Material, further inquiries can be directed to the corresponding author.
