# Supplementary material for: Molecular Modeling of ABHD5 Structure and Ligand Recognition
Source: Front Mol Biosci. 2022 Jun 28;9:935375. doi: 10.3389/fmolb.2022.935375 (PMC9274090; doi:10.3389/fmolb.2022.935375)
Supplement: Supplementary file 1 [file DataSheet1.docx]

**Supporting Information for**

**Molecular Modeling of ABHD5 Structure and Ligand Recognition**

Rezvan Shahoei^1^, Susheel Pangeni^1^, Matthew Sanders^2^, Huamei Zhang^2^, Ljljiana Mladenovic-Lucas^2^, William Roush^3^, Geoff Halvorsen^3^, Christopher V. Kelly^1^, James G. Granneman^2,4^, and Yu-ming M. Huang^1*^

^1^Department of Physics and Astronomy, Wayne State University, Detroit, MI 48201, USA

^2^Center for Molecular Medicine and Genetics, Wayne State University School of Medicine, Detroit, MI 48201, USA

^3^Department of Chemistry, The Scripps Research Institute, Jupiter, FL 33458, USA

^4^Center for Integrative Metabolic and Endocrine Research, Wayne State University School of Medicine, Detroit, MI 48201 USA

**Text S1**

We report six ABHD5 structures predicted from homology modeling and three ABHD5 protein-ligand complexes predicted from docking simulations. The structures can be downloaded from:

https://zenodo.org/record/6629888#.YqKs4mDMJ25

https://drive.google.com/drive/folders/1tfSjUP_6g_T2KEfr537_3yw4eAFjowsR?usp=sharing

**Table S1:** The sequence alignment of ABHD5 and 11 proteins using available structures from Protein Data Bank (PDB). The length (L) of amino acid (aa) sequence of the 11 proteins is listed in column 4. The sequence identity (ID), amino acid overlap length (OL), the expect-value (EV), and the overall alignment score (S) were calculated by the alignment programs, blastp-suite of protein BLAST and LALIGN. The organism corresponding to each protein sequence is also shown in the table.

| **Ligand** | **Binding Pocket Residues** |
| --- | --- |
| SR4559 | F86, G87, F114, E179, W199, L203, A206, N211, R217, K237, Y238, S240, N255, E262, H329, Y330 |
| SR3133 | F86, G87, F114, G115, R119, A202, L203, A206, N211, P212, L213, R217, F234, K237, Y238, N255, E262, Y330 |
| SR3134 | F86, G87, F114, G115, R119, N155, W181, L203, A206, N211, P212, L213, K237, Y238, N255, E262, Y330 |

**Table S2:** List of ABHD5 residues in the ligand binding pockets.

**Figure S1:** The RMSD for C𝛼 atoms of A) the entire protein, B) the N-terminal (residues 1–52), C) the insertion region (residues 198–270), and D) the protein excluding the N-terminal and the insertion region. Note that, the y-axis is different on plot D for clarity.

**Figure S2:** Root mean square fluctuation (RMSF) of wild-type (WT) ABHD5 and its mutants. The grey highlights indicate the flexible regions in the protein.

**Figure S3:** The most representative conformation from 1-𝜇s GaMD simulations for each model. The canonical alpha helices and beta sheets are shown in red and blue, respectively. The N-terminal (1–52) is shown in pink with the first insertion helix, 𝛼1, highlighted in magenta. The insertion helices 𝛼2, 𝛼3, 𝛼4, 𝛼5, and 𝛼6 are shown in orange, yellow, green, cyan, and light purple, respectively.

**Figure S4:** GaMD snapshots of Models 1, 4, 5, and 6. The positions of R299, G328, and D334 of each model are shown in A, B, C, and D. The potential polar interactions are displayed by red dash lines. The positions of E41 and R116 of each model are shown in E, F, G, and H. The canonical α helices, β sheets, and insertion α helices are shown in blue, red, and cyan, respectively. The N-terminal peptide (resides 1-33) (magenta) is close to E41 and R116 in Models 1 and 6.


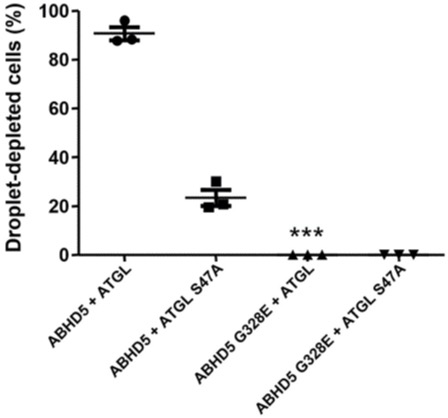


**Figure S5:** Double-blind scoring reveal that G328E ABHD5 is unable to activate ATGL through the increased abundance of LDs with cells expressing G328E vs wild-type ABHD5 (*** p < 0.001), with positive controls of lipolysis defective ATGL-S47A.

**Figure S6:** GaMD snapshots of wild-type ABHD5 and two mutants. The electrostatic interaction network (red dash lines) is shown in the wild-type ABHD5. However, K38, E41, K54, and R116N are not oriented in the same direction in the ABHD5 mutants.

**Figure S7:** Correlation maps for 1-𝜇s GaMD trajectories of ABHD5 wild-type (left) and E41A mutant (right). The purple boxes highlight the correlation among the eight 𝛽 strands for each system.

**Figure S8:** The alignment of the wild-type ABHD5 and E41A mutant. The insertion helices of the wild-type (cyan) and E41A (magenta) show different conformations in GaMD simulations. The canonical α helices and β sheets are shown in blue and red, respectively.

**Figure S9:** GaMD snapshots of the wild-type ABHD5 and G328E mutant. The detailed interactions of R299, G328/G328E, and D334 are shown in A and B. A pocket formed by the insertion helices, α2 and α4, R299, G328, and D334 is detected in the wild-type ABHD5 (C), however, the pocket closes after the G328E mutation (D). The sphere representations in C and D show R299, G328/G328E, and D334.


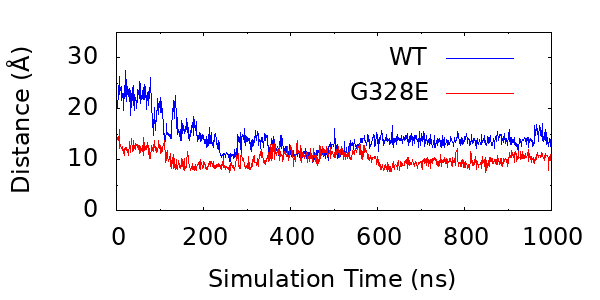


**Figure S10:** The distance between V198Cα and G328Cα/E328Cα for the wild-type (WT) ABHD5 and the G328E mutant calculated over the 1-𝜇s GaMD trajectory.

**Figure S11:** Root mean square fluctuation (RMSF) for the wild-type (WT) ABHD5 in apo and three ligand-bound conformations calculated over 1-𝜇s GaMD trajectory for each system.
